# Removing non-nuclei information from histopathological images: A preprocessing step towards improving nuclei segmentation methods

**DOI:** 10.1016/j.jpi.2023.100315

**Published:** 2023-04-18

**Authors:** Ricardo Moncayo, Anne L. Martel, Eduardo Romero

**Affiliations:** a Computer Imaging and Medical Applications Laboratory (CIM@LAB), Universidad Nacional de Colombia, Bogotá, Colombia; b Department of Medical Biophysics, University of Toronto, Ontario, Canada; c Physical Sciences, Sunnybrook Research Institute, Toronto, Ontario, Canada

**Keywords:** Cancer disease, Histopathology, Nuclei detection, Noiselet transformation

## Abstract

Disease interpretation by computer-aided diagnosis systems in digital pathology depends on reliable detection and segmentation of nuclei in hematoxylin and eosin (HE) images. These 2 tasks are challenging since appearance of both cell nuclei and background structures are very variable. This paper presents a method to improve nuclei detection and segmentation in HE images by removing tiles that only contain background information. The method divides each image into smaller patches and uses their projection to the noiselet space to capture different spatial features from non-nuclei background and nuclei structures. The noiselet features are clustered by a *K*-means algorithm and the resultant partition, defined by the cluster centroids, is herein named the noiselet code-book. A part of an image, a tile, is divided into patches and represented by the histogram of occurrences of the projected patches in the noiselet code-book. Finally, with these histograms, a classifier learns to differentiate between nuclei and non-nuclei tiles. By applying a conventional watershed-marked method to detect and segment nuclei, evaluation consisted in comparing pure watershed method against denoising-plus-watershed in an open database with 8 different types of tissues. The averaged F-score of nuclei detection improved from 0.830 to 0.86 and the dice score after segmentation increased from 0.701 to 0.723.

## Introduction

Cancer is a group of diseases characterized by an uncontrolled division of abnormal cells, which eventually invade and induce abnormal processes in other tissues, and contribute to a significant number of global deaths.^1^ Diagnosis is basically achieved by microscopic analysis, a complex process for which a tissue sample is extracted from the body, fixated and stained to reveal particular patterns. Hematoxylin and eosin (H&E) is the most used stain since it provides a broad spectrum of cellular and extracellular components.^2^ Generally, the subjective grading systems used for estimating disease aggressiveness are prone to errors^3^ and depend on manual measures which are imprecise, time-consuming, and unrealistic in daily practice.^4^

Digital pathology has emerged as a promising approach to overcome these challenges. Digitized versions of the whole slide (WSI) are used for remote diagnosis, education, and Computer Aid Diagnosis systems (CADs).^5^ CADs aim to quantify structural relationships associated with the disease, including the tumor microenvironment,^6^ to reduce the existing degree of diagnosis uncertainty by analyzing a considerable number of cellular and extracellular components.^5^

Nuclei segmentation is often a crucial component of the analysis pipeline and is highly dependent on a variety of factors such as proper fixation, sample orientation within the paraffin block, staining protocols, and tissue heterogeneity. Every organ requires a precise protocol for any of these steps and therefore any deviation or slight change introduces unpredictable noise in the pipeline. The diagnosis process may also be affected by blurring regions in the whole slide caused by tissue folds and bubbles.^7^ Most nuclei segmentation methods rely on the relative image intensity values, i.e., finding blob-like structures at different image intensities. Some of these methods include watershed markers, vector support machines, neural networks, and regression models.^6^ Usually, these methods intend to separate H&E stains and regularize them to improve nuclei and non-nuclei representations as a pre-processing step, but definitely an effective pre-processing needs to improve the signal-to-noise ratio, or in other words to remove what is not nuclei from the image.

**Fig. 1:**
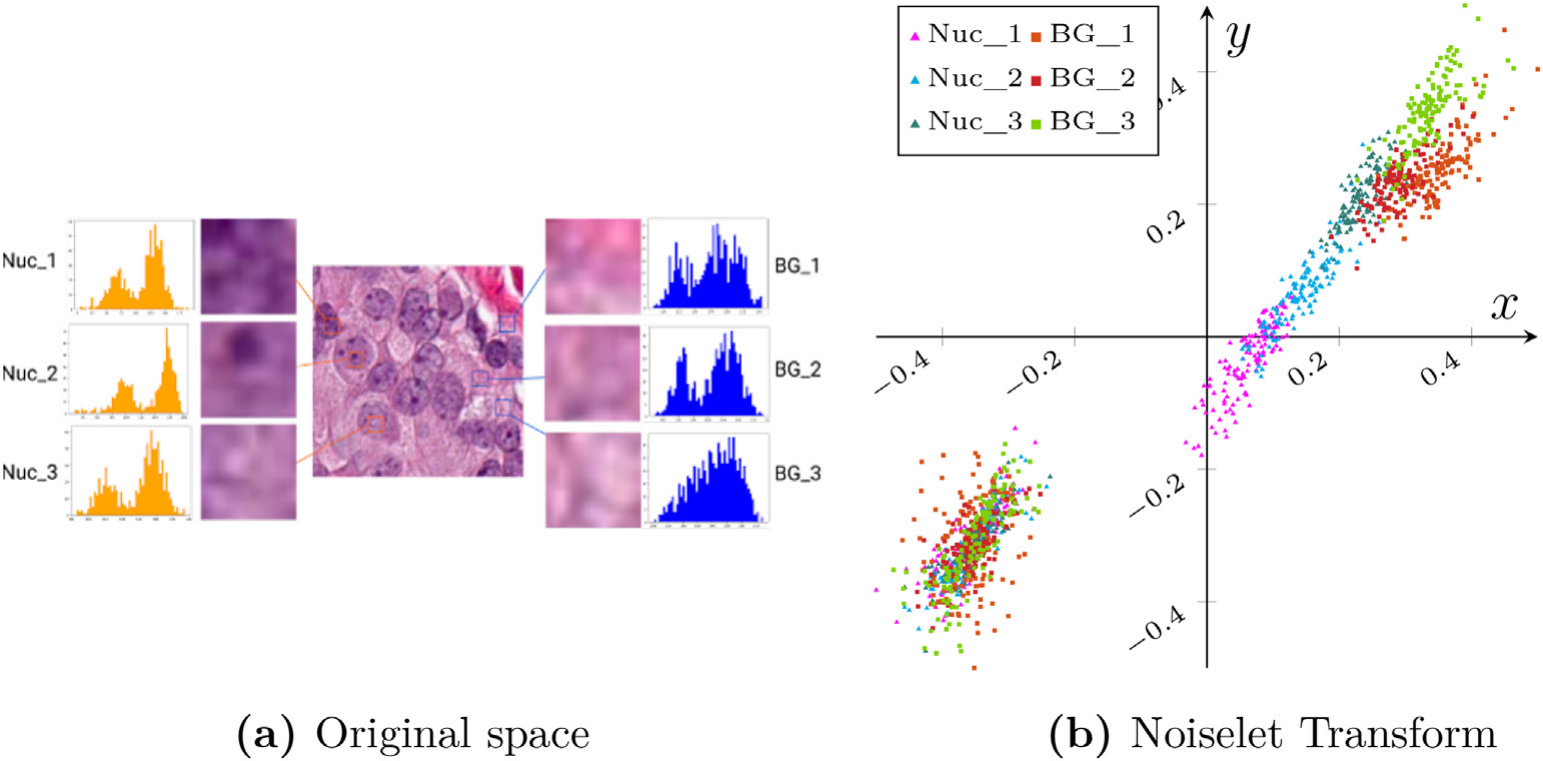
The left part of Fig. 1a shows the histograms of nuclei (left column) and non-nuclei (right column) of patches of 16×16 pixels. Typically, nuclei patches exhibit a normal or bi-modal distribution, while non-nuclei structures show a much flatter spectrum. Notice how these distributions resemble each other and could be easily confused in the image space, however a very different picture is observed in the noiselet space Fig. 1b, where the complex plane of the representation clearly displays the 2 classes into separate locations.

This work focuses on characterizing non-nuclei signals, removing them, and improving detection of the remaining nuclei. The broad signal spectrum of non-nuclei signals (Fig. 1a) may limit any characterization and many terms would be necessary to approximate this signal. Instead, this noise is represented by a family of multi-resolution functions with a great incoherence between low and high frequencies, the noiselet family, which achieves a natural sparse non-nuclei representation (Fig. 1b). Once signals are projected to the noiselet space, a classifier learns how to differentiate nuclei from non-nuclei information, i.e., particular spatial patterns related with stroma, tissue foldings, fibrous structures, and background. This method demonstrated to be robust with different types of tissue and color variations and significantly improved nuclei segmentation and detection.

## Related work

Most methods identify nuclei-related blobs using a combination of techniques like color deconvolution, thresholding, morphology operations, region growing, and machine learning algorithms such as support vector machines or convolutional neural networks.^6^^,^^8^ Recently, some researchers have pointed out the need of characterizing the background, cytoplasm, or stromal tissue, to improve both nuclei detection and segmentation.^9^ However, few studies have used non-nuclei characterization as part of their methodologies.

Some strategies have aimed to enhance hematoxylin-eosin contrast and improve segmentation accuracy. Peikari et al.^10^ transform the color space before segmenting nuclei by applying a decorrelation stretch, which equalizes the color variance in a space generated by the 3 largest eigenvectors of the covariance matrix obtained from the RGB image. Nuclei are then segmented by the otsu’s threshold followed of some morphological operations. This method was assessed at detecting nuclei in a database with 36 H&E images and 7931 manually segmented nuclei, including breast, liver, gastric mucosa, and bone marrow specimens, reporting a mean F-score of 0.901. Hou et al.^11^ proposed a sparse auto-encoder for unsupervised nucleus detection along with background and foreground separation. The auto-encoder reconstructs an image using a series of feature maps capturing low resolution background information. The difference between the original image and the reconstructed background is then used to generate a foreground image. An F-score of 0.834 was reported detecting nuclei.

Feng et al.^12^ proposed a stacked denoising autoencoder neural network for nuclei and non-nuclei classification. The first encoder–decoder architecture finds a coding representation of the image and then a corrupted version is used as input of a second encoder–decoder, being the output a denoised version of the input image, minimizing a cost function based on the Kullback–Leibler divergence. Once the architecture was trained, the output of the second encoder architecture was used to feed a softmax to classify between nuclei and non-nuclei patches of 16×16 pixels. The proposed method was evaluated on 2 breast cancer datasets, 1 containing malignant tissue and the other with only benign tissue, reporting a F-score of 0.901 and 0.97 respectively for a classification task.

## Proposed methodology

This work aims to improve the nuclei signal from several tissues by subtracting wide spectrum noise. The strategy starts by constructing a set of customized noiselet bases obtained from first-order Hadamard functions (i.e., patches of 4×4 pixels) which are clustered by a *k*-means algorithm using a city-block distance. This clusterization of the noiselet space (the code-book), as illustrated in Fig. 3, constitutes a basic vocabulary. A color deconvolution technique converts the input image into hematoxylin and eosin channels.^13^ The hematoxylin image is decomposed into tiles (i.e., 16×16 pixels) which are represented as a frequency histogram of the noiselet code-book. A simple classifier separates the nuclei from non-nuclei tiles which are removed from the image, as illustrated in Fig. 4.

### Code-book construction

#### Hematoxylin image

**Fig. 2:**
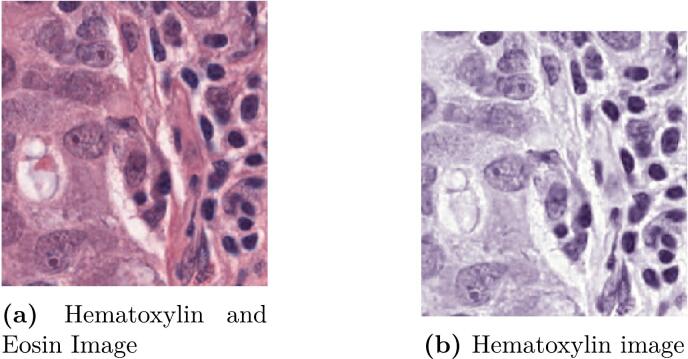
The figure presents a histological image and the hematoxylin representation after the color deconvolution procedure. Hematoxylin image conserves non-nuclei components.

The color deconvolution approach proposed by Macenko et al.^13^ is used to separate Hematoxylin and eosin stains of the image, i.e, non-nuclei and nuclei signals are characterized. Nevertheless, this low level separation works at the level of pixel and therefore considerable non-nuclei signal is observable in the hematoxylin image, as shown in Fig. 2.

**Fig. 3:**
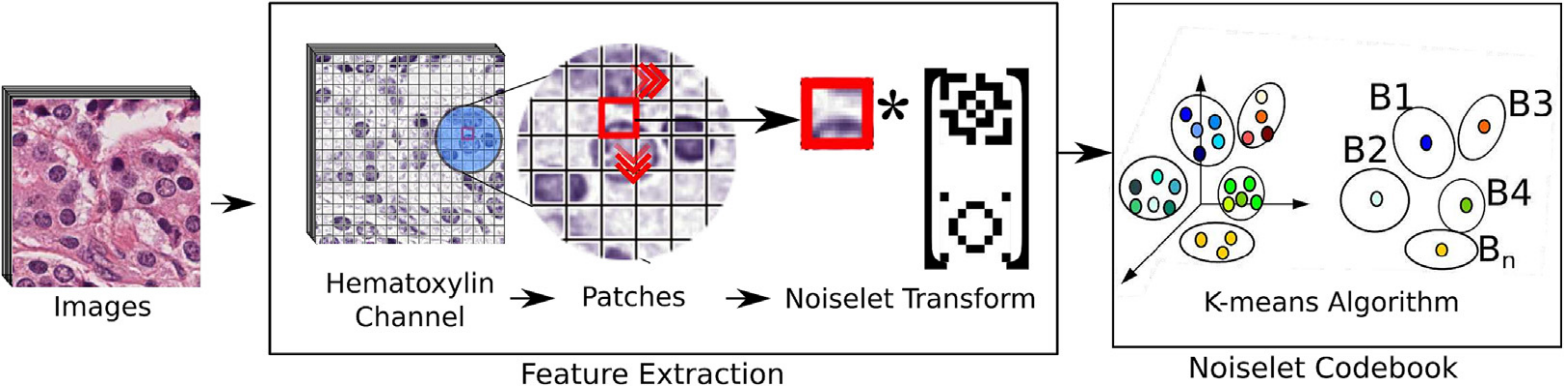
Code-book Construction: the hematoxylin (H) color channel of the input image is split into small patches. They are transformed to the noiselet space which is clustered using a *k*-means algorithm, being each cluster a coded word of the nuclei or non-nuclei signals.

**Fig. 4:**
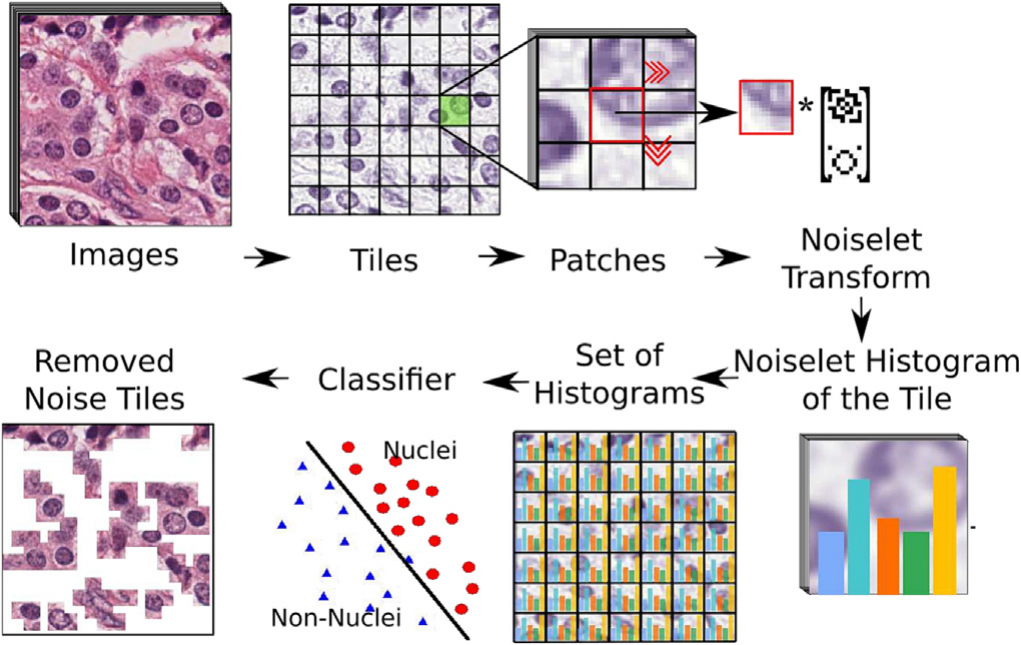
Any image may be represented as a set of histograms obtained from a tile decomposition of the input image (i.e., 12×12). Basically, an image is split into tiles and each tile is divided into patches to be projected to the noiselet space. The obtained noiselet coefficients are quantized by computing the nearest word of the noiselet code-book. A per-tile-histogram counts the number occurrences of every tile word in the code-book. A set of training images, characterized by their histograms, train a binary classifier to discriminate nuclei and non-nuclei signals. Tiles classified as non-nuclei signal are removed from the image.

**Fig. 5:**
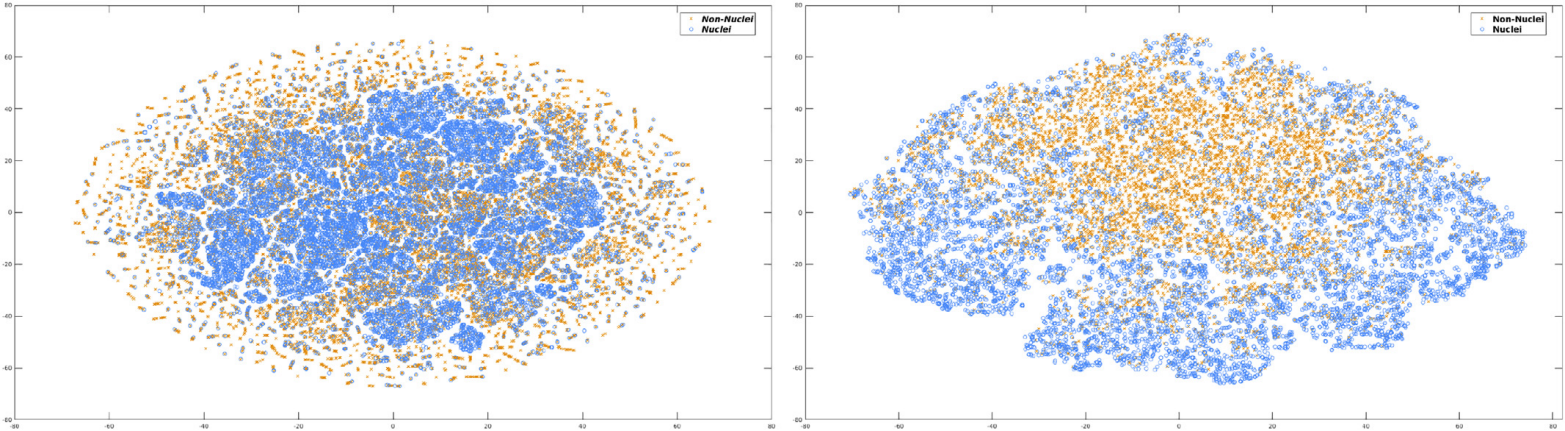
Noiselet coefficients from 2×2 and 4×4 tiles, projected to a plane by a t-distributed stochastic neighbor embedding (*t-SNE*). left panel shows the projection of 2×2 tiles while the right panel displays the projections of 4×4 tiles. Blue circles correspond to nuclei signal and orange crosses to non-nuclei ones.

**Fig. 6:**
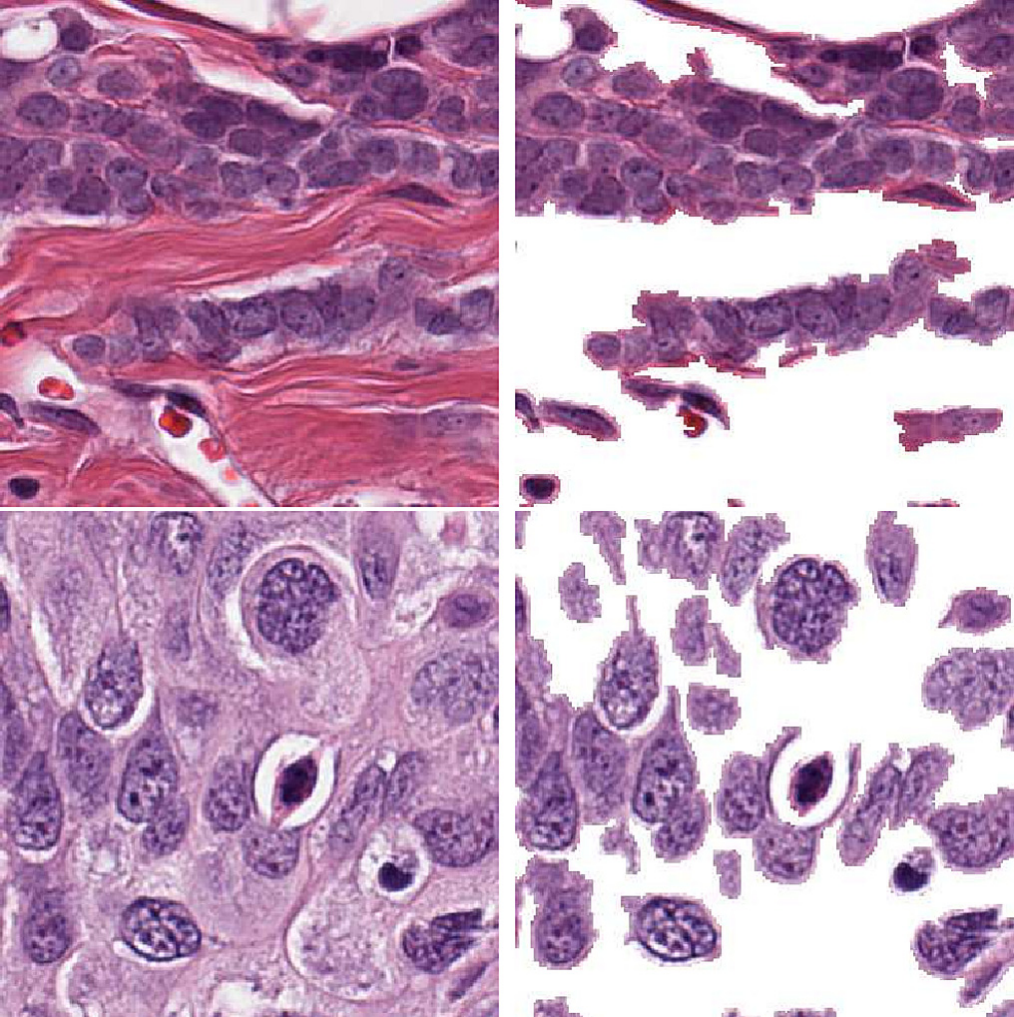
Non-nuclei signal removal in 2 different images. The left column corresponds to input images and the right column the resulting image after removing noise with the proposed methodology.

#### Extraction of patches

A hematoxylin tile is decomposed into non-overlapping patches, which are transformed into the noiselet space. Their coefficients are associated with code words of a code-book constructed by a simple *K*-means clustering of the whole noiselet space. The patch size is a parameter dependent on the analyzed tissue. In section results, it will be shown this parameter is optimal in patches of size 2×2 and 4×4 pixels for most of the assessed tissues.

#### Noiselet transform

The crucial problem to be addressed here is how to describe a variety of structures that together comprehend a wide spectrum of frequencies, even though each of them covers narrow frequency bands. Complex textures and patterns are often characterized using a large number of statistical terms but this is a problem for classification algorithms where performance may drop as the number of features increases, a phenomenon known as the “curse of dimensionality”.^14^ An alternative is to approximate the frequency spectrum with few coefficients, a primary characteristic of a specific family of wavelet functions: the noiselets. Generally, any wavelet family performs the frequency analysis by projecting an input to orthogonal low and high-frequency sub-spaces, yet a certain degree of overlapping is always unavoidable. Overall, this effect, observed at the analysis step, is compensated by complementary filters in the synthesis step that removes such aliasing. From the family of wavelets, the noiselets are unique as they project low and high frequencies to separated quadrants of the complex plane, aiming to decrease the analysis overlapping, i.e., different analysis phases.^15^ This property facilitates to cover the whole frequency spectrum with a minimum number of scales or orthogonal sub-spaces.

Noiselets are functions constructed from any wavelet family, trying to make the low and high pass filters as incoherent as possible. In this work, noiselets are built from the Haar-Walsh wavelet family.^15^ Unlike traditional wavelet analysis, noiselet families can sparsely capture information from the whole spectrum, a property that makes them ideal to characterize several types of tissue, i.e., nuclei, stroma, hyperplasic blood vessels, or particular cellular organizations.

In practice, even and odd noiselet coefficients are obtained by dyadically projecting the low-frequency information to the (1-*i*), (1+*i*) quadrant, while high-frequency information is mapped to the complement, the (1+*i*), (1-*i*) quadrant. This separates low and high frequencies by a phase of 90 in the complex plane, achieving maximum frequency decoupling. As with the Wavelet Transform, Noiselets require data length to be a power of 2.

(1) $$\begin{array}{c} f_{1}\left(x\right)=\chi_{\left[01\right)}\left(x\right)\\ f_{2n}\left(x\right)=\left(1-i\right)f_{n}\left(2x\right)+\left(1+i\right)f_{n}\left(2x-1\right)\\ f_{2n+1}\left(x\right)=\left(1+i\right)f_{n}\left(2x\right)+\left(1-i\right)f_{n}\left(2x-1\right) \end{array}$$

In summary, each patch to be analyzed is projected to a noiselet base, constructed after Eq. (1). The low pass noiselet function *f*_1_(*x*) = *χ*_[0,1)_(*x*) defines the indicatrix function, a heavy side function over the subset [0,1). The high pass functions, *f*_2*n*_(*x*) and *f*_2*n*+1_, are the projections of the Walsh functions to different quadrants of the complex plane. An illustration of the effect of this transformation is presented in Fig. 1b. This figure shows in the complex plane the noiselet coefficients obtained from patches of 16×16 pixels (see Fig. 1a. Interestingly, while nuclei and background histograms look quite similar, the noiselet transform provides a separation between these 2 classes in quadrants I and IV.

#### The noiselet code-book

A region of interest is herein defined by the nucleus size, i.e., typically from 20×20 to 70×70 pixels. A small image square (2×2 pixels), the patch, is chosen as the information unit to be projected to the noiselet space (8 possible frequencies). Different tissues and structures, i.e., stroma, nuclei, or blood vessels, are mapped to the noiselet space and the resultant coefficients are clustered to construct a noiselet vocabulary, the code-book. Basically, the noiselet coefficients are grouped by a traditional *k*-means algorithm under a city–block distance since the computational time is smaller. This feature space partition serves to represent any input image as a histogram in the noiselet space. For doing so, the image is split into patches of the typical size and mapped to the noiselet space to be assigned to the nearest code in the code-book.

### Learning the noise representation

Any field of view is divided into non-overlapping tiles (12×12 pixels) which are split into small patches (4×4 pixels). Each patch is projected to the noiselet space and the obtained set of coefficients is mapped to the noiselet code-book, i.e., each noiselet coefficient is assigned to the nearest word in the code-book. Finally, a histogram per tile is obtained by counting the number of word occurrences in the code-book. The obtained noiselet features per class are learned by an Adaboost classifier, a set of weighted weak classifiers which are combined to construct a robust classifier.^16^

## Evaluation and results

### Data

Experiments were performed in the MoNuSeg dataset for nuclei segmentation of the MICCAI conference 2018 challenge.^17^ This dataset is composed of 45 fields of view at 40× magnification (1000×1000 pixels), contain images from 8 different tissues with more than 21 000 manually annotated nuclei. The dataset was built by randomly selecting data from the Cancer Genome Atlas (TCGA)^18^ and the selected fields of view were manually annotated by trained students supervised by a pathologist. Each field of view comes from 9 different diseases: breast invasive carcinoma, kidney renal papillary cell carcinoma, prostate adenocarcinoma, bladder urothelial cancer, colon adenocarcinoma, stomach adenocarcinoma, lung adenocarcinoma and squamous cell carcinoma, and brain lower grade glioma.

A brief description of these pathologies is hereafter introduced to illustrate the variety of noises characterizing them.

**Breast invasive carcinoma:** Three different types are identified, lobular invasive breast cancer in around 10%, invasive ductal breast cancer in about 80%, and a remaining 10% with other tumors.^19^ In addition, 5 different histological variants have been identified, classic type, pleomorphic lobular carcinoma, histiocytoid carcinoma, signet ring carcinoma, and tubulo lobular carcinoma.^19^

**Brain lower grade glioma (LGG):** This tumor comprehends more than a dozen of distinct entities with unique clinicopathological, radiographic, and genetic features. glial cells are subdivided in astrocytes, ependymal cells, and oligodendroglial cells.^20^^,^^21^

**Kidney renal papillary cell carcinoma:** Two types are described. Type 1 - Papillae are lined with a single layer of basophilic cells, with scanty basophilic cytoplasm and low nuclear grade, and Type 2 - Papillae are aligned with pseudostratified layers of cells of higher nucleolar grade.^22^^,^^23^ Actually morphological features overlap and the subtypes are practically in-determined using current image techniques.

**Prostate adenocarcinoma:** Low grade patterns correspond to separated and well-formed glands with single malignant glands and nuclear enlargement, while higher grades are identified by clusters of malignant glands with stromal infiltration and cribriform glands, and nuclear enlargement with prominent nucleoli is present.^24^

**Bladder urothelial carcinoma:** Urothelial carcinoma may be in situ or invasive. The in situ carcinoma is characterized by cytologic cells with marked atypia that replaces the urothelium. The urothelial carcinoma is invasive when neoplastic cells invade the bladder wall as nest, trabeculae, small clusters, or single cells usually separated by a desmoplastic stroma.^25^

**Colon adenocarcinoma:** Nine subtypes of colon cancer are recognized, adenoma, adenosquamous carcinoma with sarcomatoid components, medullary, micropapillary, mucinous, serrated, signet ring cell, undifferentiated.^26^ Tumor cells are continuous with the serosal surface and isolated cancer cells may be presented in lymph nodes and micrometastasis.^27^

**Stomach adenocarcinoma:** The intestinal type of gastric cancer is often related to environmental factors such as *Helicobacter pylori* infection, diet, and lifestyle. The diffuse type is more often associated with genetic abnormalities. Gastric carcinoma has marked heterogeneity at the architectural and cytologic level, 4 major histologic patterns are recognized, tubular papillary, mucinous and poorly cohesive, and uncommon histologic variants.^28^

**Lung adenocarcinoma:** Five invasive tumor patterns, or variable combinations, may be identified: Adenocarcinoma with lepidic growth, cells proliferating in the surface of the alveolar walls without stromal or vascular invasion,^29^ the papillary pattern in which papilar structures replaces the alveolar architecture,^30^ or the micropapillary arrangement with micro-papillae without a fibrovascular core, and the solid type with mucin.^31^

**Lung squamous cell carcinoma:** Squamous cell carcinoma is subdivided into keratinizing and non-keratinizing tumors, the former being characterized by the formation of horn pearls and intercellular bridges or keratinization.^32^^,^^33^ The cells are arranged in sheets or nests, are polygonal with moderate to abundant eosinophilic cytoplasm, have well-defined intercellular borders, and a nucleus that may contain vesicular chromatin or be hyperchromatic. Keratinized cells show a dense eosinophilic cytoplasm with small hyperchromatic nuclei.^33^ These characteristics vary with the degree of differentiation: The higher the degree, the more prominent these characteristics are.^32^^,^^33^ Non-keratinizing cases present nests of polygonal cells that require immunohistochemistry to determine their lineage, given their morphological similarity to large-cell carcinoma. Diffuse squamous markers (p40, p63, CK5/6) and TTF1 negativity confirm its squamous phenotype.^32^^,^^33^

### Evaluation

Parameter setting: An Adaboost model is trained for each tissue and validated using a leave-one-out scheme. The 3 model parameters: the patch size, the tile size, and the number of clusters for the code-book construction, were found by exhaustive search in each tissue, after a total of 52 experiments. The parameter setup is presented in Table 1.

**Table 1:** Experimental parameter setup.

	Values
Patch size	2×2, 4×4, 8×8
Tile size	8×8, 12×8, 16×16, 20×20, 24×24
Codes	2, 4, 8, 16

#### Nuclei detection and segmentation task

This work was evaluated by comparing the performance of a classic nuclei segmenter before and after removing non-nuclei signals. The nuclei segmentation method herein applied^34^ starts by obtaining a hematoxylin image from a color deconvolution of the original image,^13^ and detecting nuclei candidates in this channel by a fast radial transform along with a marker-controlled watershed algorithm. Finally, morphological filters removed regions not complying with the particular area, roundness, and solidity criteria.

Nuclei segmentation performance is computed as the Dice score between the obtained segmentation (*A*) and the ground truth (*B*) (Eq. 2). In addition, the F-score evaluates nuclei detection after Eq. (3), being a true positive (TP), a detected nucleus intersecting the ground truth with a distance between centroids smaller than 12 pixels, otherwise detection is a false positive (FP). Likewise, a FP is a detected nucleus corresponding to stroma region or a nucleus with no intersection with a ground truth.

(2) $$\mathrm{DSC}\left(A,B\right)=\frac{2|A\cap B|}{|A|+|B|}$$

(3) $$F1_{\mathrm{score}}=\frac{\mathrm{TP}}{\mathrm{TP}+\frac{1}{2}\left(\mathrm{FP}+\mathrm{FN}\right)}$$

#### Correlation between annotated and found nuclei

Pearson correlation analyses were performed to determine how the proposed method could eventually remove actual nuclei. Taking the available set of annotated patches, a Pearson test established correlation between the number of annotated nuclei and the number of nuclei remaining after the method was applied.

#### Evaluation with a deep learning approach

The method was also assessed as a preprocessing of a pre-trained neural network, a HoVer-Net architecture^35^ with the weights available from the authors in the MonuSeg dataset. Breast and colorectal cancer datasets (Table 2)^36^^,^^35^ were segmented by applying this neural network on a particular image or segmenting the same image pre-processed by the proposed method. Both segmentations were then compared.

**Table 2:** Dataset used to compare segmentations by the HoVer-Net neural network and this network plus pre-processing.

Tissue	Breast TNBC	Colorectal CoNSeP
Images	50	41
Magnification	40×	40×
Resolution pixels	1000×1000	1000×1000
Number of Nuclei	4022	24 319

### Results

In the first part of this section, the results of nuclei segmentation and detection with optimal parameters of each tissue are presented for both the proposed method and the baseline. The second part presents a cluster analysis of the noiselet description between nuclei and non-nuclei

#### Nuclei segmentation metrics

Notice this improvement in Table 3 is quite variable because of the noise nature. For the brain low-grade glioma (LG), detection improvement is 0.035 in F-score, yet a slight dice score variation is observed (-0.003). Stomach adenocarcinoma, lung adenocarcinoma, breast invasive carcinoma, and colon adenocarcinoma tissues show comparable improvements (F-score difference >0.03, dice-score difference >0.028). For prostate adenocarcinoma tissue, a minor improvement is observed, i.e., F-score difference is 0.018 and nuclei segmentation is enhanced in 0.025. Kidney exhibit a F-score difference larger than 0.021 and nuclei segmentation enhancement of 0.018. Finally, the bladder urothelial carcinoma tissue shows a nuclei detection improvement in F-score of 0.027.

**Table 3:** Results for MONUSEG dataset.

Tissue	Patch size	Tile size	Codes	F-score baseline	F-score method	Dice-score baseline	Dice-score method	Nuclei correlation
Bladder urothelial carcinoma	2×2	16×16	8	0.838	**0.865**	**0.741**	0.742	0.967
Brain lower grade glioma	4×4	16×16	16	0.836	**0.871**	**0.729**	0.726	1.00
Breast invasive carcinoma	2×2	12×12	8	0.819	**0.850**	0.691	**0.721**	0.932
Colon adenocarcinoma	2×2	24×24	4	0.788	**0.825**	0.644	**0.684**	0.944
Kidney renal papillary cell carcinoma	4×4	8×8	8	0.843	**0.866**	0.720	**0.738**	0.9861
Lung adenocarcinoma	4×4	12×12	4	0.810	**0.849**	0.653	**0.693**	1.000
Prostate adenocarcinoma	2×2	24×24	16	0.788	**0.806**	0.636	**0.661**	0.869
Stomach adenocarcinoma	2×2	12×12	16	0.906	**0.946**	0.794	**0.822**	1.000
Average				0.830	**0.860**	0.701	**0.723**	0.962

#### Analysis of clusters of noiselet patches in two-dimensional space

Overall, the noiselet feature space offers a clear separation between nuclei and non-nuclei signals, as illustrated in Fig. 5, which displays a 2-dimensional *t-SNE* projection of the noiselet coefficients for the 2 classes. The plot illustrates the level of separation between the 2 classes, with each occupying distinct regions of the noiselet space. The result of non-signal elimination for 2 different patches is presented in Fig. 6. The left panels show the original patches in the noiselet space, while the right panels display the same patches after removing non-nuclei signals. These 2 examples show removal of stroma in upper panel and elimination of interstitial tissue in the bottom panel.

#### Correlation analyses results

Correlation tests were run between the number of annotated nuclei and the number of nuclei detected after application of the proposed methodology. A high correlation (1.00) was observed in brain, lung, stomach, and colon tissues, while kidney and bladder respectively showed a correlation of 0.962 and 0.965, and breast and prostate tissues of 0.78 and 0.74.

These results strongly suggest the proposed method eliminates a small proportion of actual nuclei in almost all tissues, even in breast and prostate tissues, demonstrating the method improves nuclei detection in different tissues, as presented in the previous subsection.

#### Results on a deep learning approach

Results in Table 4 show the proposed method increases both the F-score and Dice score metrics, the former indicating nuclei detection is improved while the latter suggesting actual nuclei were easier to find after pre-processing, all simply applying a pre-trained HoVer-Net architecture. For the colorectal dataset, the F-score increased by 0.018 and the Dice score by 0.037, while for the breast cancer dataset, these figures corresponded to 0.01 and 0.043.

**Table 4:** Nuclei detection results with the CoNSeP and TNBC datasets using the proposed method and a deep learning architecture.

Dataset	Colorectal CoNSeP	Breast TNBC
F_score	**0.592**	**0.832**
Proposed+Hovernet		
F_score	0.574	0.822
Hovernet		
Dice score	**0.677**	**0.798**
Proposed+Hovernet		
Dice Score	0.640	0.755
Hovernet		

## Discussion

Any automatic cancer analysis or diagnosis definitely depends on the quality of nuclei segmentation. This work focuses in removing non-nuclei signals in hematoxylin and eosin images. Briefly, a discretized noiselet space is obtained by mapping small image patches (typically 2×2 pixels) labeled as nuclei or non-nuclei and performing a clusterization in the feature space. This discrete feature space, the code-book, allows comparisons at the level of the probability distribution of data by estimating distances between histograms of occurrences of these codes in larger tiles (typically 12×12 pixels). In fact, these histograms feed an AdaBoost classifier which learns to discriminate between non-nuclei and nuclei signals.

Unlike previous methods that remove non-nuclei signals, based on hematoxylin-eosin separation, morphological operations, or color thresholds, the proposed method characterizes and eliminates a broad spectrum of non-nuclei signals (noisy background) by mapping them to a customized noiselet space, i.e., different regions of the compex plane. Peikari et al.^10^ improved nuclei detection by stretching the RGB image and projecting it to the 3 largest variance eigenvectors, followed by a classic equalization. This strategy was tested in 4 organs, breast, liver, stomach, and bone marrow, from a single institute with the same acquisition protocol, reporting a mean F-score of 0.91. The presented work, evaluated in stomach samples from 6 different institutes and breast adenocarcinomas, obtained a F-score of 0.946 and 0.850 for 2 different institutions. Likewise, Kumar et al.,^17^ with different CNN architectures, reported a mean F-score of 0.826 for detecting nuclei in the MonuSeg dataset (the same collection herein tested) and the presented method outperformed this detection with a mean F-score of 0.860.

Despite the intrinsic nuclei and connective tissue variabilities, the presented approach improved the performance of a classic watershed segmenter,^34^ specifically for 8 different abnormal tissues. The optimal experimental parameters, i.e., patch size, tile size, and number of codes, vary with the tissue under analysis. In 5 tissues, bladder, breast, colon, prostate, and stomach carcinomas, the patch size with better results was 2×2 pixels, suggesting that a single noiselet scale describes the main frequency differences between signal and noise. On the other hand, in brain, kidney, and lung adenocarcinoma, the patch size with better performance was 4×4 pixels, likely because these tissues exhibit a wider stroma spectrum and more noiselet scales are necessary to separate both signals. In these cases, the tile size varied, i.e., while better results were observed in tiles with 12×12 pixels for breast and colon, for stomach cancer the best results were obtained with tiles of 24×24 pixels, and for bladder and brain carcinoma, 16×16 pixels. Overall, the tile size was less important since the obtained F-scores around the optimum (3) were very similar, even though the number of possible frequencies is determined by the tile size, v.g., breast and stomach cells exhibits more circularity when compared with bladder ones which are pyramidal, or with prostate and colon whose cells are elongated.

Overall, when grouping the noiselet space with a simple *k*-means for only 2 codes, each group contains at least a >90% of the signal, i.e., while one group is basically composed of nuclei, the other cluster contains non-nuclei signal (see Fig. 6). Nevertheless, this is not completely true for all tissues since their vriability influences the optimal number of codes required to obtain a proper detection. Typically, the non-nuclei signal demands a larger number of codes to be represented, and this number may be even larger when several non-nuclei tissue types are present. In particular, colon adenocarcinoma was represented by 4 codes, from which 3 represented mostly the non-nuclei and the remaining one, the nuclei signal. In case of lung cancer, 2 codes stood for nuclei and 2 non-nuclei signal. In breast, kidney, and bladder adenocarcinomas, the optimal number of codes was 8, in breast tissue 2 codes contained most of the nuclei signal, in kidney 3 codes and 2 codes in bladder. Finally, prostate, stomach, and brain adenocarcinomas were represented by 16 codes, and while in prostate and brain tissues, 2 codes were basically composed of nuclei signal, in stomach the same signal was represented by 3 codes. These findings indicate the more tissue variability is observed the larger the number of codes to characterize and separate both signals.

A strong correlation was observed between the number of detected and annotated nuclei in a variety of different tissues, indicating the method consistently removed non-nuclei information. A particular experiment demonstrated this method can be easily integrated with state-of-the-art nuclei detectors, the output of the method was used as input to a pre-trained neural network architecture and both F-score and Dice-score improved, indicating integration of the 2 approaches is not only possible but rather complementary. Future research should investigate the impact of removing non-nuclei signals on the general training pipeline of neural networks.

## Conclusions

This work presents a novel method to characterize and remove noise or non-nuclei signals from actual histopathology samples. Non-correlated patterns can be described by the noiselet transform to improve the performance of any nuclei segmenter. This proposal achieves competitive results in detecting and segmenting nuclei in eight different tissues. Data in the simplest noiselet space show different space locations for nuclei and stromal information. Future works will integrate this methodology within a complete segmentation pipeline.

## Declaration of interests

The authors declare that they have no known competing financial interests or personal relationships that could have appeared to influence the work reported in this paper.
